# Construction and validation of a prognostic model for gastric cancer patients with tumor deposits

**DOI:** 10.7717/peerj.17751

**Published:** 2024-07-10

**Authors:** Ran Xu, Yisheng Zhang, Zhengguang Wang, Ke Chen, Jun Zhao

**Affiliations:** 1 Department of General Surgery, The Yijishan Hospital of Wannan Medical College, Wuhu, China; 2 Department of General Surgery, The First Affiliated Hospital of Anhui Medical University, Hefei, China; 3 Department of Vascular Surgery, Drum Tower Hospital, Jiangsu, China

**Keywords:** Stomach tumor, Tumor deposits, Prognosis, Adjuvant chemotherapy, Nomogram

## Abstract

**Background:**

Tumor deposits (TD) was a significant risk factor impacting the prognosis of patients diagnosed with gastric cancer (GC), yet it was not currently incorporated into TNM staging systems. The objective of this research was to develop a predictive model for assessing the prognosis of patients with TD-positive GC.

**Methods:**

Retrospective analysis was performed on the data of 4,972 patients treated for GC with D2 radical gastrectomy at Wannan Medical College’s Yijishan Hospital between January 2012 and December 2021. The patients were categorized based on the number of TD (L1: 1, L2: 2–3, L3: ≥4) and the anatomical location of TD (Q1: single area, Q2: multiple areas). In a 3:1 ratio, patients were randomly assigned to one of two groups: training or validation.

**Results:**

The study included a total of 575 patients who were divided into the training group (*n* = 432) and validation group (*n* = 143). Survival analysis showed that the number and anatomical location of TD had a significant impact on the prognosis of patients with TD-positive GC. Univariate analysis of the training group data revealed that tumor size, T-stage, N-stage, histological grade, number and distribution of TD, neural invasion, and postoperative chemotherapy were associated with prognosis. Multivariate Cox regression analysis identified poor histological grade, T4 stage, N3 stage, number of TD, neural invasion, and postoperative chemotherapy as independent prognostic factors for GC patients with TD. A nomogram was developed using these variables, demonstrating well predictive ability for 1, 3, and 5-year overall survival (OS) in the validation set. The DCA curve shows that the constructed model shows a large positive net gain compared to the eighth edition Tumour, Node, Metastasis (TNM) staging system.

**Conclusion:**

The prognostic model developed for patients with TD-positive GC has a higher clinical utility compared to the eighth edition of TNM staging.

## Introduction

The incidence and mortality of gastric cancer (GC) have been decreasing due to advancements in medical technology and increased awareness of health. However, it remains a leading cause of cancer deaths, especially in Asian countries like China, Japan, and Korea (Sung, Ferlay & Siegel, 2021). The prognosis and treatment strategies for GC patients primarily rely on the Tumour, Node, Metastasis (TNM) stage of the cancer (Amin, Edge & Greene, 2017). In China, the lack of an early screening system often leads to the diagnosis of advanced GC. Consequently, the standard treatment for these patients typically involves radical surgery followed by postoperative adjuvant chemotherapy (Association Japanese Gastric Cancer, 2021). However, it is worth noting that individuals with identical TNM stage may experience differing prognoses despite receiving identical treatment. Researchers from different countries have actively investigated prognostic factors for GC and have found that factors such as neural invasion *(Li et al., 2020)*, histological differentiation (specifically ‘signet ring cell’) (Xie et al., 2022), microsatellite status (Miceli et al., 2019; Cristescu et al., 2015), and tumor deposits (TD) (Etoh et al., 2006; Wang et al., 2011; Sun et al., 2011; Gu et al., 2020; Lee et al., 2013; Fujikawa et al., 2023) may impact the prognosis of GC patients.

Tumor deposits are a neoplastic nodule that is discontinuous with the primary lesion and lacks identifiable lymphoid tissue structures. It is distributed in the lymph node drainage area of the lesion (Amin, Edge & Greene, 2017; Wang et al., 2011). The detection rate of TD has gradually increased due to advancements in surgical laparoscopic instruments, membrane anatomy techniques, and pathological detection techniques. Studies have reported a detection rate ranging from 6.3% to 23.9% (Lee et al., 2013; Fujikawa et al., 2023). Liang et al. (2019) reported that the TD detection rate of GC radical specimens was even as high as 27.5% (Liang et al., 2019). Numerous studies (Etoh et al., 2006; Wang et al., 2011; Sun et al., 2011; Gu et al., 2020; Lee et al., 2013; Fujikawa et al., 2023; Liang et al., 2019) have confirmed that TD predicts poor prognosis in patients undergoing surgical treatment for GC. However, TD in GC was not currently included in the TNM staging system and was only used as a registration indicator (Amin, Edge & Greene, 2017). In colorectal cancer, in the absence of lymph node metastasis, TD has been treated as an N1c stage (Amin, Edge & Greene, 2017) . The eighth edition of the TNM staging system cannot effectively assess the prognosis of patients with TD-positive GC. This study seeks to investigate how the number and distribution of TD affect the prognosis of TD-positive GC patients. The goal is to develop a prognostic prediction model that can assist clinicians in assessing the prognosis of this disease.

## Patients and Methods

### Study population

**Figure 1:**
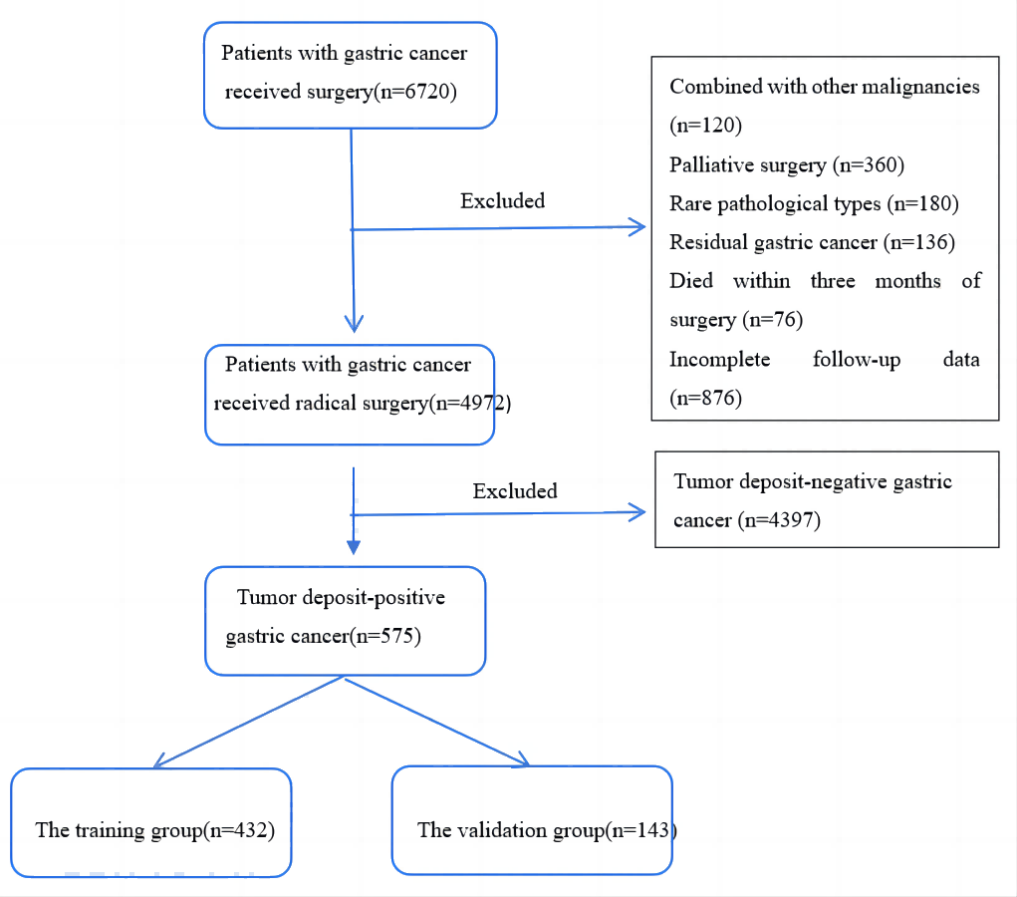
A flow chart of patient inclusion.

We conducted a study on GC patients who underwent surgery at Yijishan Hospital of Wannan Medical College between January 2012 and December 2021. The study included patients who met the following inclusion criteria: (1) underwent standard D2 radical surgery for GC (Association Japanese Gastric Cancer, 2021), (2) did not receive preoperative radiotherapy or chemotherapy, (3) had no distant metastases before or during the operation, (4) had postoperative pathological reports confirming gastric adenocarcinoma with TD, (5) had more than 16 lymph nodes dissected, and (6) had complete follow-up data. We excluded patients who met the following exclusion criteria: (1) had other primary tumor diseases, (2) underwent emergency palliative surgery due to GC combined with perforation and obstruction, (3) had rare pathological types such as hepatoid adenocarcinoma or neuroendocrine carcinoma, (4) had residual GC or multifocal carcinoma, (5) died within three months of surgery, mainly due to postoperative complications such as anastomotic leakage, infectious shock, and cardiopulmonary failure. We collected clinical and pathological data for the included patients based on the 8th edition of the TNM staging system for GC by the American Joint Committee on Cancer (AJCC) and the International Union Against Cancer (UICC). The flowchart for the inclusion of patients is presented in Fig. 1. The collected data included gender, age, T stage, N stage, tumor differentiation, tumor location, number and distribution of TD, neural invasion, vascular invasion, surgical method, and postoperative chemotherapy status.

Patients who required adjuvant chemotherapy following surgery were chosen according to the 7th edition of the AJCC-TNM staging system (stage II–III). The primary adjuvant chemotherapy regimens utilized were fluoropyrimidine-based regimens, including CapeOX, SOX, S-1, or capecitabine. This study followed the ethical guidelines outlined in the Helsinki Declaration. The research project underwent thorough review and certification by the Ethics Committee of Wannan Medical College Yijishan Hospital (approval number: 2021–136). All patients gave oral informed consent before gastroscopy, surgery, or chemotherapy, and every procedure was performed according to the rules of clinical practice.

### Definition of TD, the grouping of patients based on the number and distribution of TD

**Figure 2:**
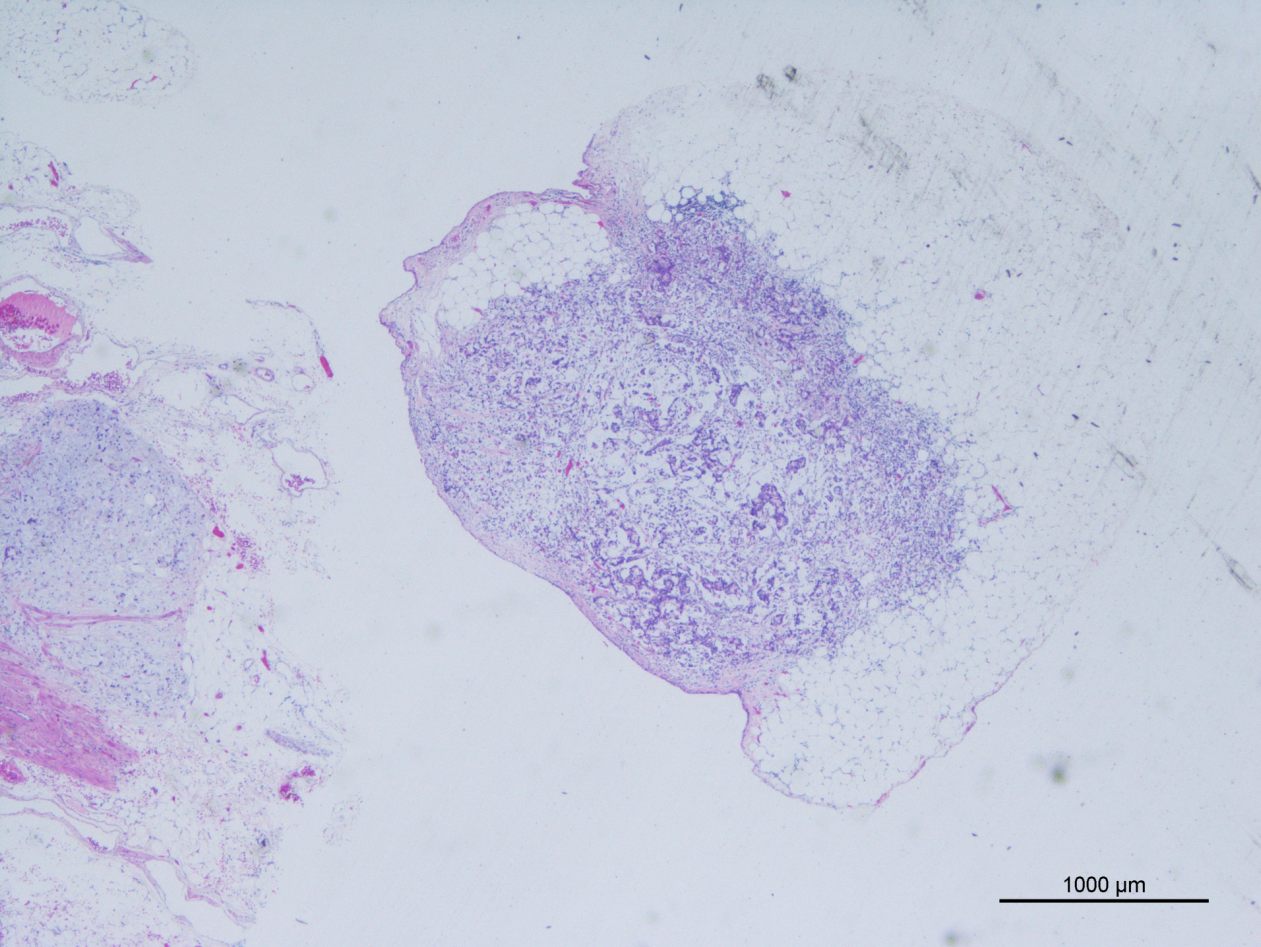
Tumor deposits (H&E, magnification ×20) cancer cells have been found in adipose tissue that is not connected to the primary lesion.

**Figure 3:**
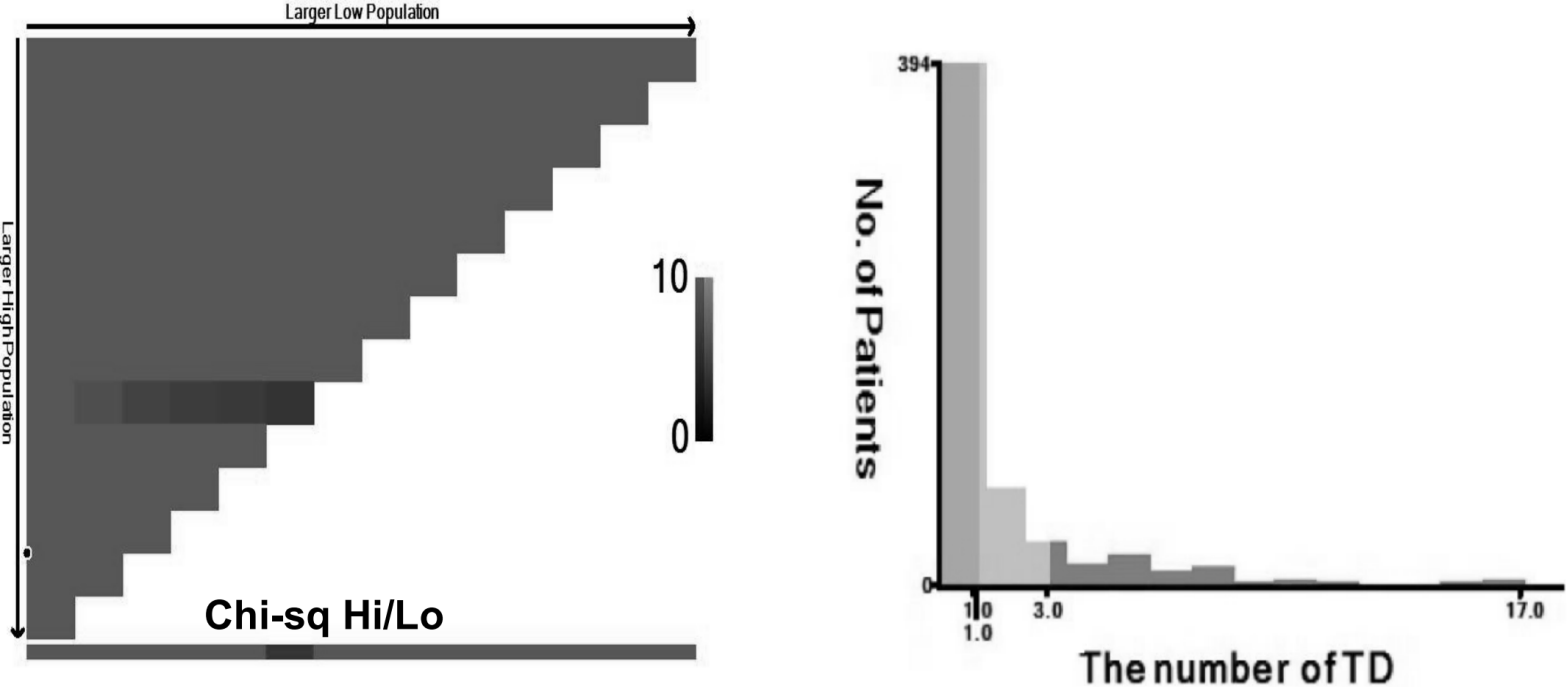
TD are grouped based on the number of TD, L1 (*n* = 1), L2 (*n* = 2–3), L3 (*n* ≥ 4).

The AJCC 8th edition for GC states that TD is an unconnected tumor nodule. It is present within the area of lymphatic drainage of the gastric primary focus and does not have identifiable structures of lymph nodes, lymphatics, nerves, or blood vessels, regardless of its morphology and size (Amin, Edge & Greene, 2017). The pathology is illustrated in Fig. 2. In this case, the original tissue sections were retrieved, and two pathologists independently assessed whether it met the criteria for TD. If there was disagreement between the pathologists, a third pathologist made the final determination. The optimal cut-off value for the number of TD was determined by analyzing the impact on overall survival time using X-tile software (Camp, Dolled-Filhart & Rimm, 2004), as depicted in Fig. 3. In this study, patients were categorized into the L1 group (one TD), L2 group (two to three TD), and L3 group (four or more TD). The anatomical location grouping of TD was based on the distribution region of lymph node groups 1#-12# in the 5th edition of the Japanese guidelines for the diagnosis and treatment of GC (Association Japanese Gastric Cancer, 2021). TD found in any one of the lymph node regions of groups 1#-12# was considered a single-region distribution (group Q1), while TD found in two or more lymph node regions was considered a multi-region distribution (group Q2).

### Follow-up

Follow-up was conducted through telephone, WeChat, and outpatient visits at regular intervals. Follow-up visits were scheduled every 3 months for the first year, every 6 months for 1–2 years, and yearly after 3 years. The follow-up process involved various assessments such as physical examination, blood count, liver and kidney functions, tumor markers (CEA, Ca199, *etc*.), chest and abdominal CT, and gastroscopy. The overall survival (OS) time was measured from the date of surgery until the endpoint of follow-up or the patient’s death. The median follow-up time was 21 months (3–128 months), and the deadline for the visit has been set for October 31, 2022.

### Statistical analysis

SPSS 24.0 (Chicago, IL, USA) and R software (ver. 3.63) were used for statistical analyses. A statistically significant difference was considered at *P* < 0.05. The optimal cut-off values for the number of TD were determined using X-Tile software (Camp, Dolled-Filhart & Rimm, 2004). Survival analyses employed the Kaplan–Meier method, the Log-rank test for group comparison, and the Cox proportional risk regression model for univariate and multivariate prognostic analyses. Prognostic factors with *P* < 0.05 in univariate COX analysis were included in multivariate COX analysis. Patients were randomly assigned to training and validation groups using R packages at a 3:1 ratio. A nomogram was created based on data from the training group. Model performance was assessed using the area under curve (AUC) and consistency index (C-index), while the calibration curve evaluated predicted *versus* actual results. Decision curve analysis (DCA) assessed the clinical utility and net benefit of the model.

## Result

### Clinical and pathological characteristics

**Figure 4:**
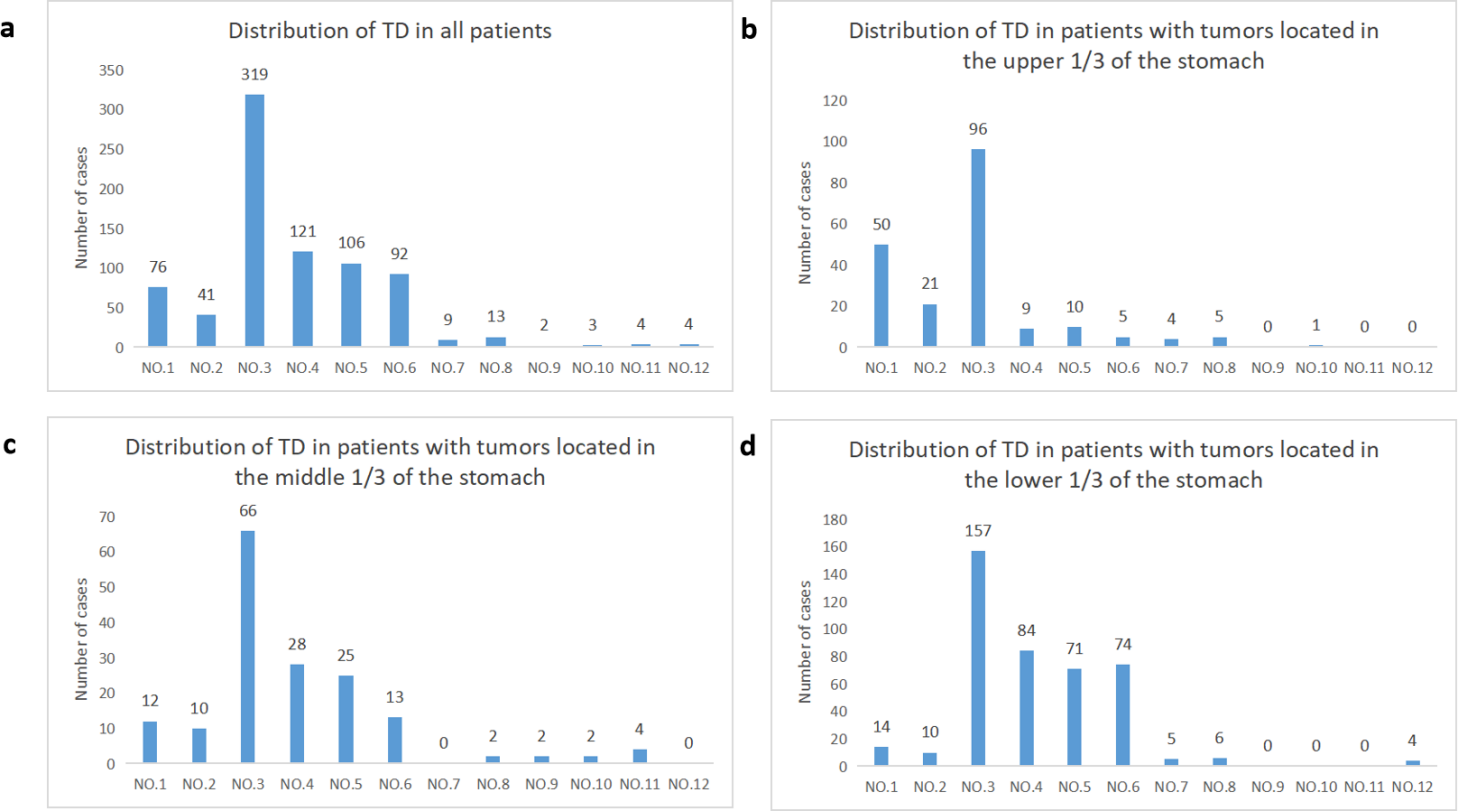
Distribution of TD in regional lymphatic drainage areas. (A) All patients, (B) patients with tumors located in the upper 1/3 of the stomach, (C) patients with tumors located in the middle 1/3 of the stomach, (D) patients with tumors located in the lower 1/3 of the stomach.

The study included a total of 575 patients, consisting of 439 males and 136 females. The median number of TD in the included cases was 2, ranging from 1 to 17. Patients were staged using TNM 8th edition, with 1.56% as stage I, 14.08% as stage II, and 84.34% as stage III. Figure 4A illustrates the distribution of TD in all cases included in the study. The probability of TD in the perigastric lymph node region (groups 1–6) was significantly higher than that in the extra-perigastric lymph node region (groups 7–12). When considering the tumor site, tumors in the upper 1/3 of the stomach showed a relatively high frequency of TD in the lesser curvature region (58.9%), left of the cardia (30.7%), and right of the cardia (12.9%) (Fig. 4B). Similarly, tumors in the middle 1/3 of the stomach had a relatively high frequency of TD in the lesser curvature region (68.8%), greater curvature region (29.2%), and supra-pyloric region (26.0%) (Fig. 4C). For tumors in the lower 1/3 of the stomach, the frequency of TD was relatively high in the lesser curvature region (51.8%), greater curvature region (27.7%), subpyloric region (24.4%), and subpyloric region (23.4%) (Fig. 4D). There is a correlation between the location of the tumor and the distribution of TD. TD is frequently found in lymphoid fat tissue near the tumor. Regardless of tumor location, the lymphatic fat in the lesser curvature region of the stomach had the highest probability of TD.

### Training group and validation group baseline data

A comparison of the 13 clinicopathological characteristics between the two groups did not reveal any statistically significant difference (*P* > 0.05) (Table 1).

**Table 1:** Clinicopathologic characteristics of all patients.

Variable	N	Training group (*n* = 432)	Validation group (*n* = 143)	*P* value
Gender				0.941
Male	439	329	110	
Female	136	103	33	
Age (years)				
<60	154	119	35	0.542
≥60	421	313	108	
Tumor size				
<5 cm	258	195	63	0.897
≥5 cm	317	237	80	
Location				0.264
Upper 1/3	163	115	48	
Middle 1/3	96	71	25	
Lower 1/3	303	237	66	
Mix	13	9	4	
T-stage				0.218
PT1	9	6	3	
PT2	35	29	6	
PT3	110	89	21	
PT4	421	308	113	
N-stage				0.713
PN0	41	31	10	
PN1	71	56	15	
PN2	152	117	35	
PN3	311	228	83	
Histologic grade				
G1/G2	398	291	107	0.116
G3	177	141	36	
Vascular invasion				
(+)	374	274	100	0.158
(−)	165	131	34	
NA	36	27	9	
Neural invasion				1
(+)	425	321	104	
(−)	130	98	32	
NA	20	13	7	
Type of gastrectomy				0.144
Proximal subtotal gastrectomy	65	43	22	
Distal gastrectomy	287	223	64	
Total gastrectomy	223	166	57	
Postoperative chemotherapy				0.990
Yes	336	253	83	
No	239	179	60	
Number of TD				0.245
L1 Group	253	182	71	
L2 Group	214	164	50	
L3 Group	108	86	22	
Distribution of TD				1
Q1 Group	432	325	107	
Q2 Group	143	107	36	

**Notes.**

G1 well- differentiated; G2 moderately differentiated; G3-poorly differentiated; NA unknown state.

### Survival analysis

#### Impact of the number and distribution of TD on overall survival in all included patients

**Figure 5:**
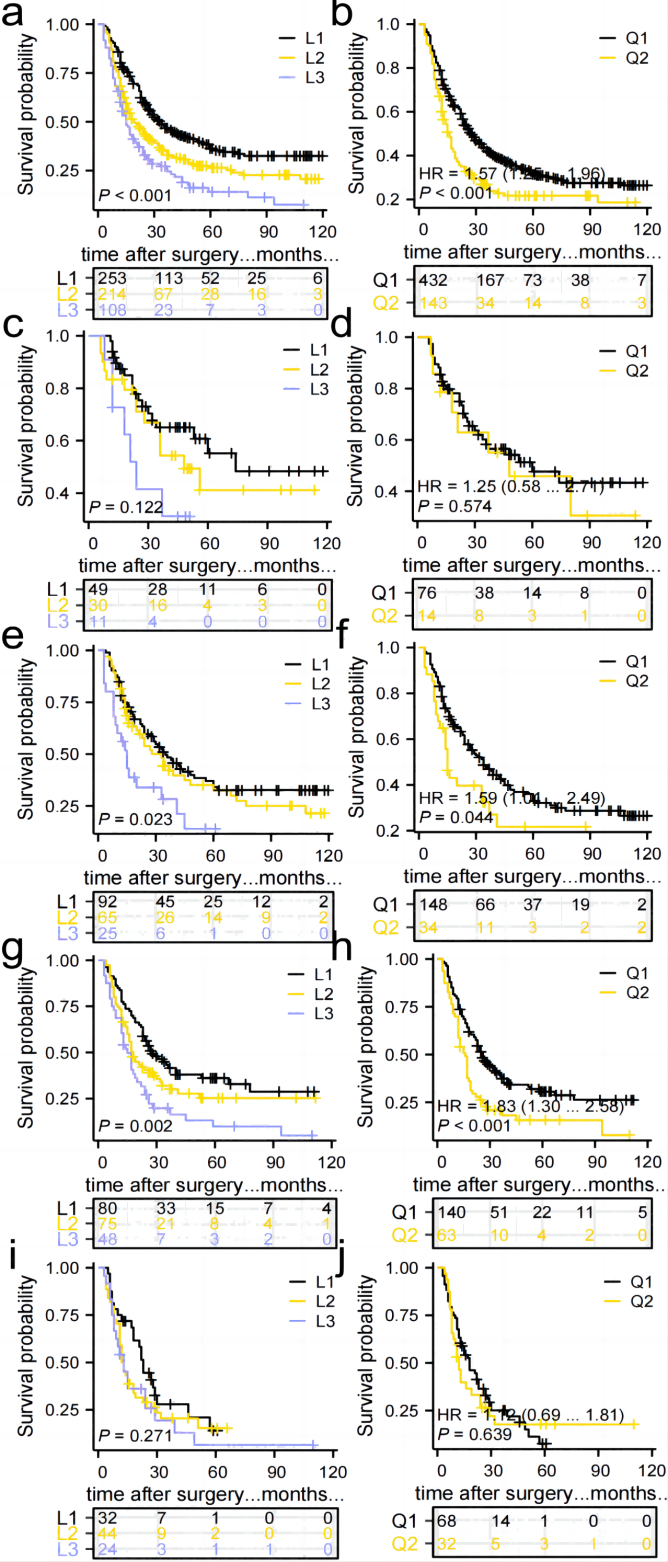
Comparison of survival curves for patients in groups L1, L2, and L3 and groups Q1 and Q2. (A, B) All patients, *p* < 0.05. (C, D)Patients in stage I–II, *p* > 0.05. (E, F) Patients in stage IIIa, *p* < 0.05. (G, H) Patients in stage IIIb, *p* < 0.05. (I, J) Patients in stage IIIc, *p* > 0.05.

The study found that the median survival time was 21 (3–128) months. The 1-year overall survival (1-OS) rates for patients in the L1, L2, and L3 groups were 80.2%, 68.2%, and 60.1% respectively. Similarly, the 3-OS rates were 46.8%, 34.8%, and 27.2% respectively, while the 5-OS rates were 36.1%, 26.4%, and 14.0% respectively. It was observed that as the number of TD increased, the 1-, 3-, and 5-OS rates decreased. Statistically significant differences in survival rates were observed between the groups (*P* < 0.05), as indicated in Fig. 5A. Furthermore, the 1-OS rates for patients in the Q1 and Q2 groups were 75.9% and 60.0% respectively, with corresponding 3-OS rates of 42.6% and 26.9%, and 5-OS rates of 31.0% and 20.5%. Notably, the prognosis of patients in the Q2 group was significantly worse than that of the Q1 group (*P* < 0.05), Fig. 5B.

### Impact of the number and distribution of TD on the prognosis of patients with stage I–II and stage IIIa, IIIb, IIIc

No significant difference in prognosis was found between the L1, L2, and L3 groups, and Q1 and Q2 groups in the Stage I–II subgroup. Therefore, the number of TD and anatomical location may not have an impact on the prognosis of Stage I–II GC patients with TD (Figs. 5C, 5D). In the Stage IIIa and IIIb subgroups, the prognosis of the L1, L2, and L3 groups showed a statistically significant difference, with the survival rate significantly decreasing as the number of TD increased. Additionally, the prognosis of the Q1 and Q2 groups differed significantly, with patients in the Q2 group experiencing a significantly worse prognosis compared to those in the Q1 group (Figs. 5E–5H). No significant difference in prognosis was observed among the L1, L2, and L3 groups in the Stage IIIc subgroup. An increase in TD did not affect the survival rate. Similarly, no significant difference in prognosis was found between the Q1 and Q2 groups (Figs. 5I, 5J).

### Prognostic impact of postoperative adjuvant chemotherapy on TD-positive GC

**Figure 6:**
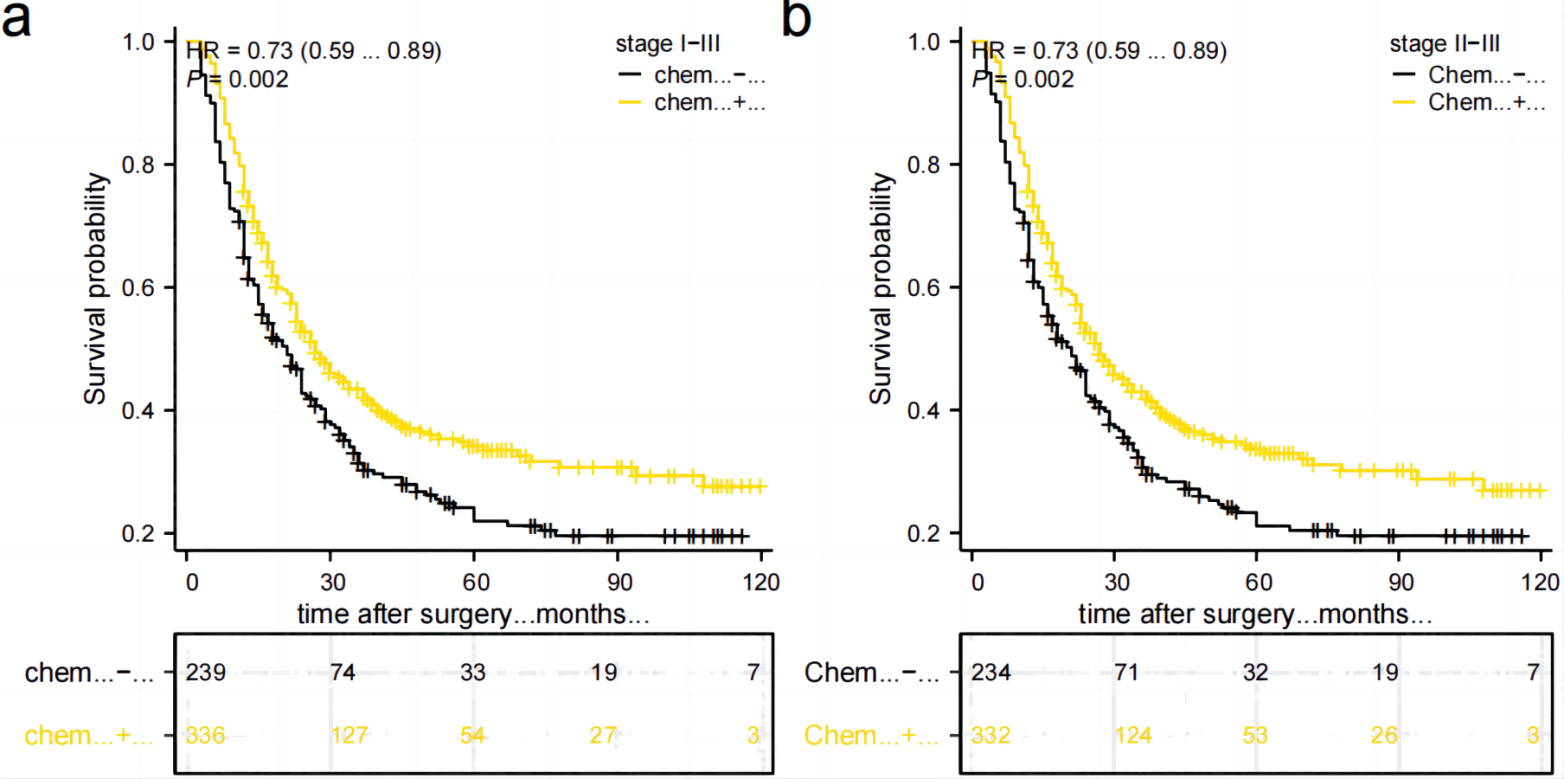
Comparison of survival curves between patients who received postoperative chemotherapy and those who did not. (A) All patients. (B) patients in stage II–III.

A total of 566 stage II–III GC patients need postoperative adjuvant chemotherapy as per Japanese guidelines (5th ed.). However, only 332 (58.6%) patients received complete standardized postoperative adjuvant chemotherapy in this study. Out of these patients, 160 (28.2%) did not adhere to the recommended treatment period of 6–8 cycles, 46 (8.1%) refused to receive postoperative adjuvant chemotherapy, and 28 (4.9%) were unable to tolerate adjuvant chemotherapy due to their physical condition. Additionally, there were nine patients in Stage I, out of which four received postoperative adjuvant chemotherapy while five did not. Comparing the postoperative chemotherapy and no-chemotherapy groups, the former had a higher 5-year OS rate than the latter (34.2% *vs* 21.9%, *P* = 0.002), Fig. 6A. Stage II–III patients who received postoperative chemotherapy had a higher 5-year OS rate than those who did not (33.6% *vs.* 21.1%, *P* = 0.002), as shown in Fig. 6B. However, due to the small number of cases in the subgroup of stage I combined with TD, it was not possible to directly compare and illustrate the benefit of postoperative chemotherapy in this specific subgroup.

### Cox regression analysis in training group

The results of the univariate analyses indicated that several factors were related to the prognosis of patients with GC accompanied by TD. These factors included tumor size, histological grade, number and distribution of TD, neural invasion, T stage, N stage, and postoperative chemotherapy. Multifactorial Cox regression analysis revealed that poor tissue differentiation, T4-stage, N3-stage, the number of TD, neural invasion, and postoperative chemotherapy were independent prognostic factors. Notably, postoperative chemotherapy was found to be a protective factor, as shown in Table 2.

**Table 2:** COX regression analyses of prognostic factors of 432 GC patients in the training group.

Characteristics	Total (N)	Univariate analysis		Multivariate analysis	
		Hazard ratio (95% CI)	*P* value	Hazard ratio (95% CI)	*P* value
Gender	432				
Male	329	Reference			
Female	103	0.869 (0.657–1.148)	0.322		
Age (years)	432				
<60	119	Reference			
≥60	313	1.143 (0.877–1.488)	0.323		
Location	432				
Mix	9	Reference			
Upper 1/3	115	0.635 (0.276–1.459)	0.285		
Middle 1/3	71	0.700 (0.300–1.636)	0.411		
Lower 1/3	237	0.534 (0.236–1.211)	0.133		
Type of gastrectomy	432				
Total gastrectomy	166	Reference			
Distal gastrectomy	223	0.849 (0.662–1.090)	0.199		
Proximal subtotal gastrectomy	43	1.158 (0.778–1.725)	0.469		
Histologic grade	432				
G1/G2	291	Reference		Reference	
G3	141	1.406 (1.104–1.791)	0.006	1.438 (1.113–1.856)	0.005
Tumor size	432				
<5 cm	195	Reference		Reference	
≥5 cm	237	1.316 (1.038–1.667)	0.023	1.101 (0.853–1.421)	0.460
T-stage	432				
T1-3	124	Reference		Reference	
T4	308	1.901 (1.409–2.564)	<0.001	1.635 (1.191–2.243)	0.002
N-stage	432				
N0-2	204	Reference		Reference	
N3	228	1.652 (1.300–2.099)	<0.001	1.388 (1.074–1.795)	0.012
Number of TD	432				
L1	182	Reference		Reference	
L2	164	1.525 (1.166–1.995)	0.002	1.430 (1.068–1.915)	0.016
L3	86	2.106 (1.548–2.864)	<0.001	1.800 (1.205–2.689)	0.004
Distribution of TD	432				
Q1	325	Reference		Reference	
Q2	107	1.552 (1.199–2.008)	<0.001	1.051 (0.758–1.456)	0.765
Neural invasion	419				
(-)	98	Reference		Reference	
(+)	321	1.711 (1.261–2.322)	<0.001	1.490 (1.086–2.044)	0.014
Vascular invasion	405				
(-)	131	Reference			
(+)	274	1.160 (0.892–1.507)	0.268		
Postoperative chemotherapy	432				
NO	179	Reference		Reference	
YES	253	0.687 (0.543–0.869)	0.002	0.655 (0.513–0.836)	<0.001

### Construction and validation of the prognostic prediction nomogram

**Figure 7:**
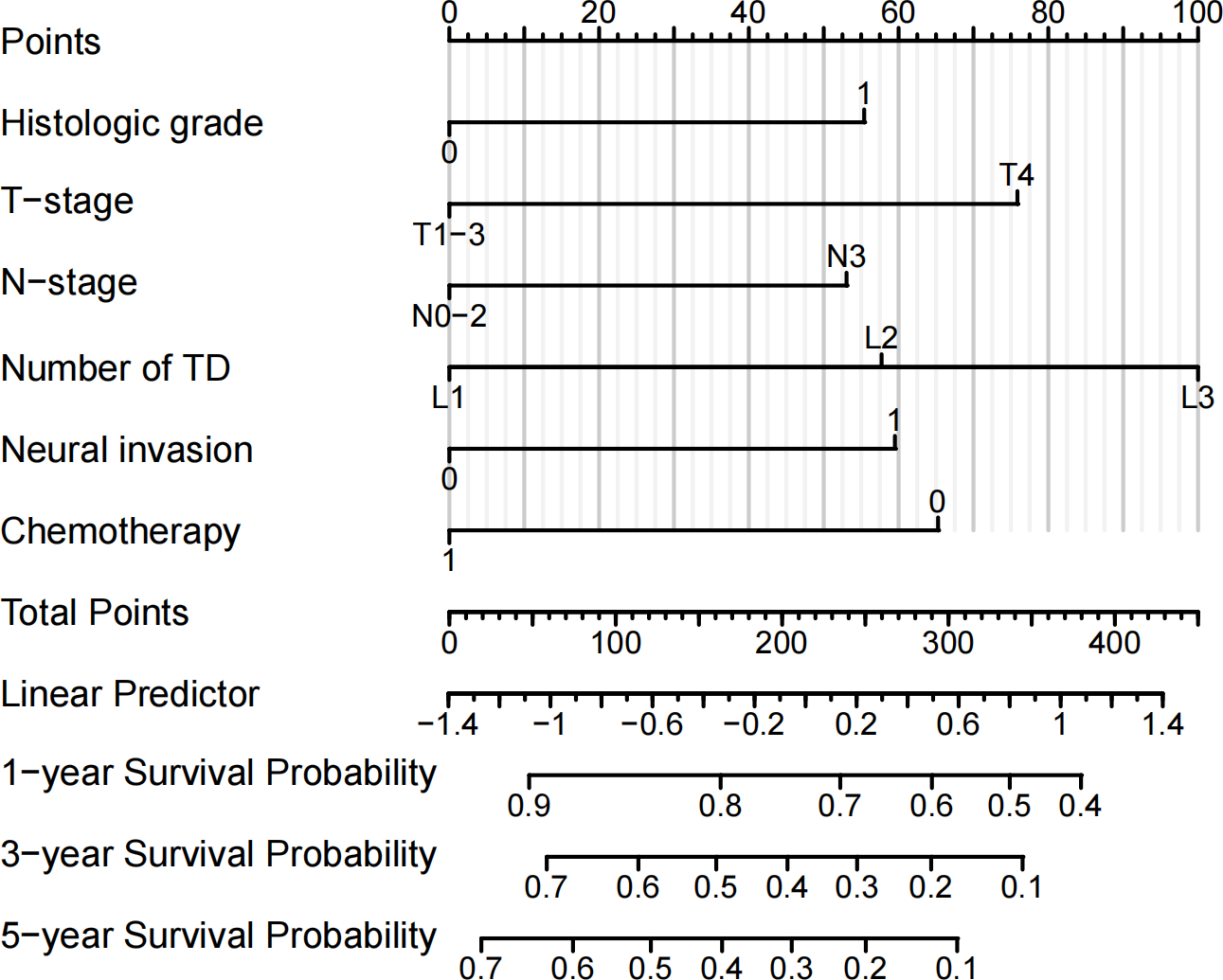
Nomogram for predicting the OS rate.

**Figure 8:**
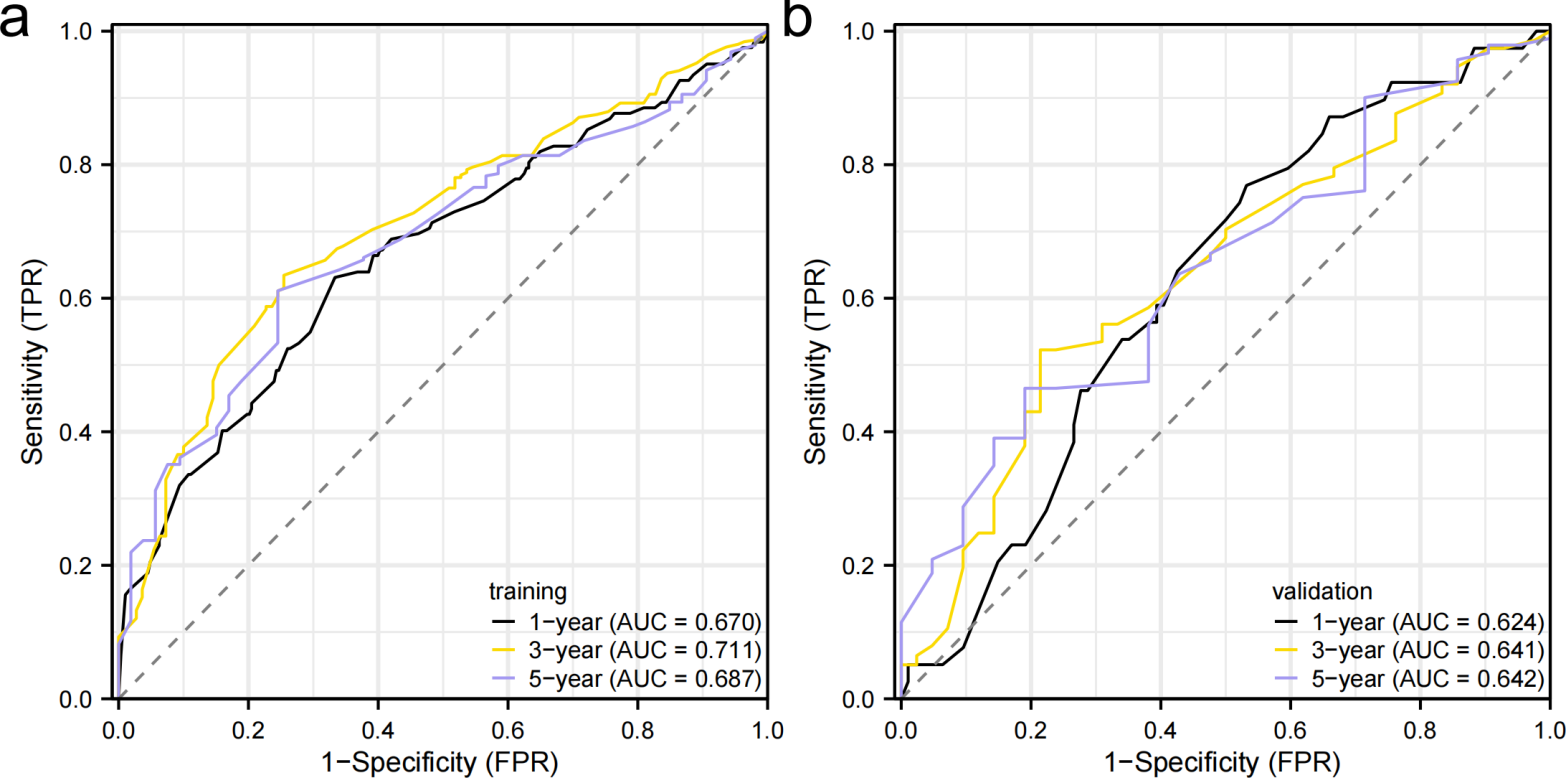
Receiver operating characteristic curves. (A) Training group. (B) validation group.

**Figure 9:**
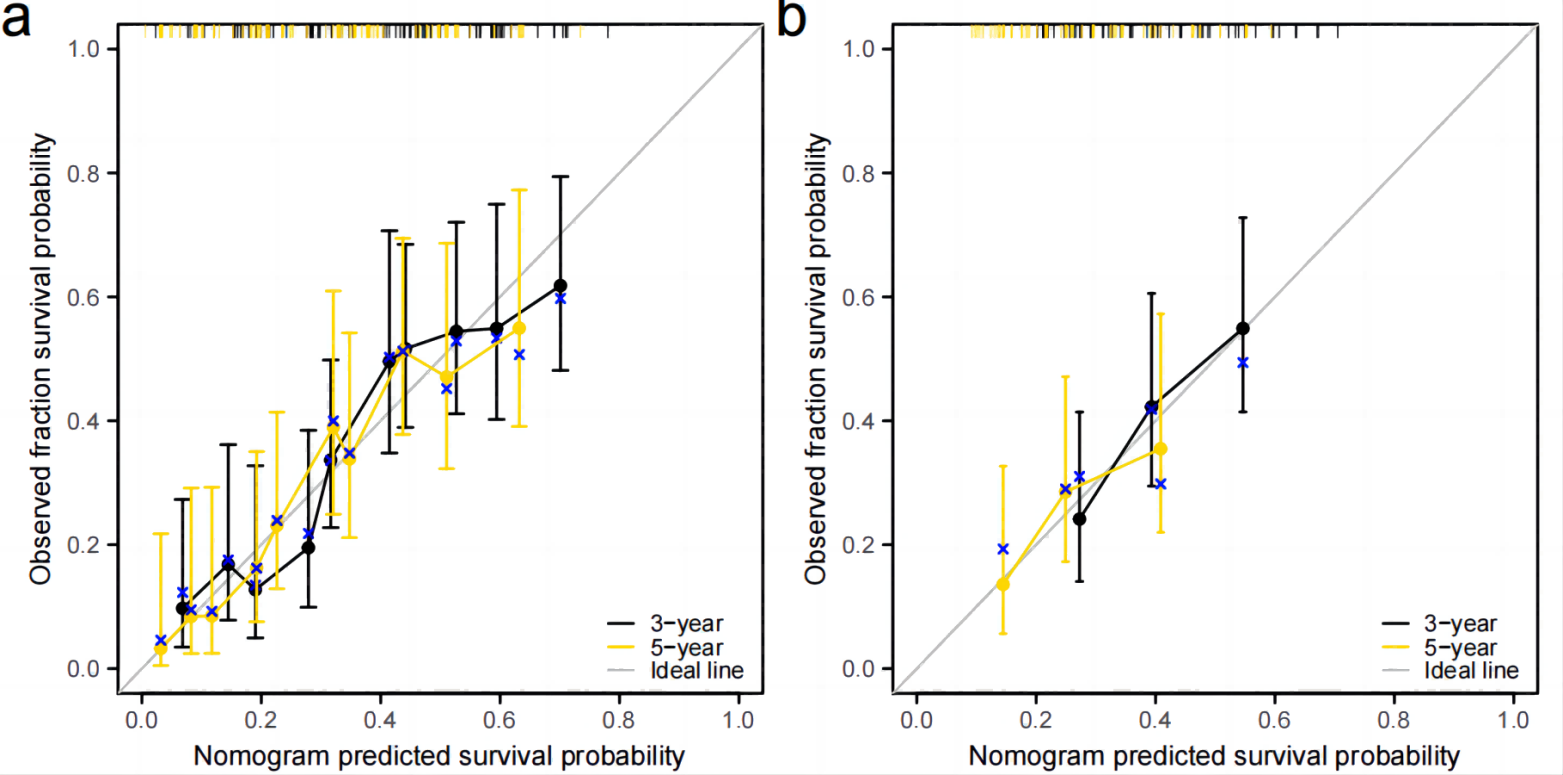
Calibration curve. (A) Training group. (B) validation group.

**Figure 10:**
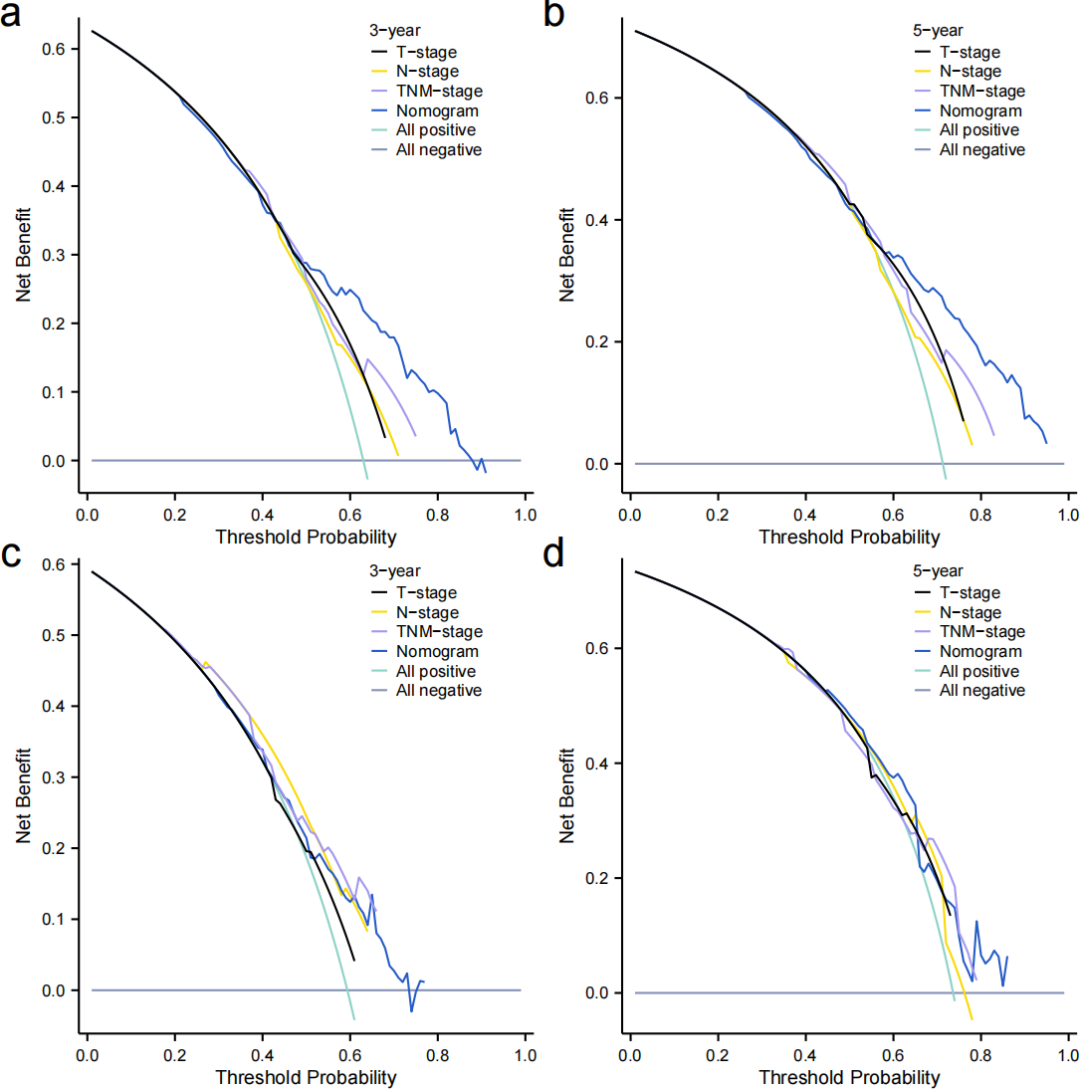
Decision curves. (A–B) Training group. (C–D) and validation group.

Independent prognostic factors identified through multifactorial Cox regression analysis were used to construct a nomogram. The nomogram allowed for the calculation of individual scores for each predictor in each patient, which were then summed to obtain a total score. The prediction of 1-, 3-, and 5-year OS rates for this group of patients was based on the probability associated with the total score on the overall scale (Fig. 7). The nomogram’s discrimination and calibration were validated using data from both the training and validation groups. The discriminatory power of the nomogram was assessed using C-Index values and ROC curves. The training and validation groups had C-indexes of 0.654 (95% CI [0.635–0.672]) and 0.616 (95% CI [0.587–0.645]) respectively for predicting overall survival rates. The nomogram’s 1-, 3-, and 5-year AUC were 0.670, 0.624, and 0.711 in the training group, and 0.641, 0.687, and 0.642 in the validation group (Fig. 8). Figure 9 displays calibration curves for predicting OS rates at 3 and 5 years for patients in the training and validation groups. Calibration curves align with the ideal line, indicating consistent predicted and actual results. Figure 10 shows DCA curves predicting survival rates for patients in training and validation groups at 3- and 5-year intervals. The DCA of the nomogram demonstrated greater net benefits compared to T-staging, N-staging, and the 8th edition TNM staging, suggesting that the nomogram offers superior clinical utility than the 8th edition TNM staging.

## Discussion

In the specific staging of GC, TD has been confirmed as a poor prognostic factor in most studies (Etoh et al., 2006; Wang et al., 2011; Sun et al., 2011; Lee et al., 2013; Anup et al., 2017). However, the mechanism of TD formation is still unknown. Most studies have shown that the appearance of TD correlates with tumor size, stage, and neurovascular infiltration, indicating high tumor aggressiveness and poor biological behavior (Sun et al., 2011; Liang et al., 2019; Huang et al., 2019; Liang et al., 2017; Yuan et al., 2023). However, there is no unified consensus on the classification and location of TD in TNM staging. Therefore, the current TNM-stage system may not be appropriate for the prognostic assessment and selection of subsequent treatment options for GC patients with TD. We conducted a thorough search for prognostic factors of GC patients with TD and developed a nomogram (Chen et al., 2022) to assist clinicians in assessing prognosis and choosing treatment plans. Therefore, this study aimed to develop an effective prognostic model for predicting the outcomes of patients with TD-positive gastric cancer and investigate the impact of the number and anatomical distribution of TD on the prognosis of these patients. The study’s findings suggest that a nomogram, incorporating histologic classification, T-stage, N-stage, number of TD, neural invasion, and postoperative adjuvant chemotherapy, can accurately predict the survival rate of patients with TD-positive GC. The model demonstrates good predictive performance and higher clinical value compared to the 8th edition of the TNM staging. Furthermore, the number of TD may impact the prognosis of patients with tumor deposits-positive GC, but it should be considered in conjunction with tumor staging. In addition, it has been established that the application of adjuvant chemotherapy after surgical procedures serves as a crucial protective factor in the prognosis of patients.

Wang et al. (2011) has demonstrated that the prognosis of patients with GC is influenced by the histologic classification, and specifically, G3-poorly differentiated is an independent risk factor that is associated with a worse prognosis. Zhang et al. (2020) analyzed the clinicopathological and prognostic data of 580 patients under the age of 70 who underwent radical gastric surgery for GC. They found that the five-year survival rate of the 34 patients in the signet ring cell-positive group was only 5.9%. Signet ring cell positivity was recognized as a distinct risk factor for this particular group of patients. Our research findings indicate that G3-poorly differentiated is a significant risk factor for patients with TD-positive GC and this is in agreement with the results of the previously mentioned study. Furthermore, among the 575 patients, 177 were classified as poorly differentiated (G3), of which 67 (37.5%) were signet ring cell-positive.

Currently, the prognosis of GC is mainly assessed based on the TNM staging. However, this staging system is slightly insufficient for the prognosis assessment of patients with TD-positive GC, as TD is not included in the staging system (Amin, Edge & Greene, 2017). Previous studies on TD (Etoh et al., 2006; Yildiz et al., 2016) have confirmed it as an independent risk factor for GC, but its attribution and positioning in the TNM staging have not been further explored. In recent years, scholars have made preliminary explorations regarding the attribution of TD in TNM staging. There are four primary viewpoints: (1) TD can be treated as metastatic lymph nodes, and the N-stage can be elevated with a positive TD status. However, there is no consensus on how to do so or the revision of the N-stage scheme. Lee et al. (2013) proposed constructing a new N staging by adding the number of cancer nodes to the number of metastatic lymph nodes, and this new pN staging has shown significant value in assessing patient survival. Chen et al. (2018) suggested adding TD positive status to N staging and upgrading N staging except for N3b. Liang et al. (2019) proposed upgrading N staging based on TD positive status, with N0 upgraded to N2, N1 upgraded to N2, and N2 upgraded to N3a, while the rest of N3a and N3b staging remained unchanged. Tan et al. (2019) believe that TD-positive status can be classified as PN3-stage. (2) TD is considered as plasma membrane infiltration that elevates T-stage to T4a staging with a positive TD status (Sun et al., 2011; Anup et al., 2017). (3) Positive TD status can directly upgrade the original pTNM-stage grading in sequence, except for stage IIIC (Gu et al., 2020; Zhou et al., 2022). Gu et al. (2020) found that the prognosis of TD(+) and TD(-) patients differed in the subgroups of stage IIA, stage IIIA, and stage IIIB. They proposed that TD(+) status could enhance pTNM-stage, except for stage IIIC. Zhou et al. (2022) found that TD was an independent risk factor for OS and DFS of patients who underwent radical surgery and validated the current 7 methods of combining TD into the TNM staging system. Finally, it was found that TD(+) status could be upgraded to TNM-stage, except for IIIC, which was the most optimal way to combine TD. The study’s findings indicated that the prognosis of patients in stage IIIa and stage IIIb subgroups was influenced by the number of TD and anatomical locations affected. However, there was no influence in stage I–II and stage IIIc subgroups. The possible reason for these findings is that the low number of stage I–II cases and the late staging of the tumor itself, along with the short prognostic survival time of the patients in the stage IIIc subgroup. Therefore, the study supports the elevation of the pTNM-stage by TD, except for stage IIIc. (4) TD should not only focus on the prognostic impact of TD status on GC but also the number of TD. Wang et al. (2011) studied the grouping of patients based on the number of TD (0, 1–2, ≥3) and found that patients with EM1 (*n* = 1–2) had a similar prognosis to patients in the pN3-stage subgroup of the TD-negative group, while patients with EM2 (n ≥3) had a similar prognosis to patients in the pM1-stage subgroup of the TD-negative group. Therefore, it is considered that the prognosis of GC patients with TD is influenced by the number of TD. Jiang et al. (2014) grouped the number of TD as EM0 (*n* = 0), EM1 (*n* = 1), EM2 (n ≥2), and then merged EM1 and EM2 into N-stage respectively. They also divided the original four grades of pN (0, 1, 2, 3) into six grades of pNE (0, 1, 2, 3, 4, 5). The new pNE staging was found to have a better prognostic predictive ability than the original 7th version of the pN stage. Zhang et al. (2020) categorized TD groups by the number (0, 1–3, ≥4) and observed differences in the 8th edition pTNM stage among the IIA, IIB, IIIA, IIIB, IIIC subgroups for the E0 group (*n* = 0), E1-3 group (*n* = 1-3), and E4 (n ≥4) group. They proposed integrating the number of TD into the pTNME staging system and emphasized that TD should be considered as an independent prognostic factor separate from T, N, and M factors. The pTNME classification may reduce staging migration and provide more accurate survival predictions for patients with GC containing TD. The impact of the number of TD on GC prognosis remains controversial. One reason is the lack of a standardized grouping criterion for TD. Additionally, there may be inconsistencies in the subjects included in different studies. In this study, we utilized X-tile software to analyze the effect of TD numbers on the overall survival time of TD-positive GC patients. The optimal cut-off value for TD was determined as (1, 2–3, ≥4), and it was observed that the prognostic survival curves worsened with an increase in the number of TD. Furthermore, in the training set data, multifactorial COX regression analysis revealed that the number of TD was an independent risk factor for these patients. These findings suggest that the number of TD significantly impacts GC prognosis, emphasizing the need to consider factors beyond the TD status alone.

There have been fewer studies investigating the effect of the anatomical location of TD on GC prognosis. Tonouchi et al. (2019), a Japanese scholar, classified patients with node-positive GC into perigastric (#1-#7 group) and non-perigastric (#8-#12 group) groups. They found that the prognosis of patients with TD located in the non-perigastric area was significantly poorer than those with TD in the perigastric area group. Therefore, it is suggested that clinicians should pay more attention to non-perigastric cancer nodes and consider necessary auxiliary treatments. In our study, we observed that the probability of TD being distributed in the perigastric lymph node region (groups 1#-6#) was significantly higher than in the lymph node region outside the perigastric area (groups 7#-12#). We also found a correlation between the distribution of TD and tumor location, as they were often found in lymphoid fat tissue closer to the tumor. Additionally, the probability of TD in the lymphoid fat in the region of the lesser curvature of the stomach was the highest, regardless of the tumor location. Understanding these patterns can aid in the diagnosis, staging, and management of gastric cancer patients. Univariate COX regression analysis revealed that the multi-regional distribution of TD was a factor for poor prognosis in patients with TD-positive GC. However, this factor did not show significant results in multivariate analyses and was not included in the final nomogram model. The results of most studies (Zhou et al., 2022; Li & Yang, 2022) have shown that neural invasion is a correlate of TD formation in GC. Neural invasion was positive in 73.9% (425/575) of the cases in our study. Poor prognosis for GC patients is associated with neural invasion, consistent with previous research findings (Wang et al., 2011; Zhi et al., 2019).

Appropriate postoperative adjuvant chemotherapy following curative resection can improve survival rates for advanced GC patients, particularly those with stage II–III disease. Postoperative adjuvant chemotherapy aims to control residual tumor cells after curative resection and reduce recurrence (Takahari et al., 2011; Bang et al., 2012). However, the current guidelines do not provide clear regulations regarding whether stage I GC patients with TD after surgery should undergo adjuvant chemotherapy. Lee et al. (2018), a Korean scholar, conducted a study on prognostic factors for stage IB GC and found that TD is a risk factor for these patients. They suggested that these patients should receive adjuvant chemotherapy after surgery. Similarly, Zhi et al. (2019) investigated the predictive significance of TD in gastric cancer patients without lymph node involvement. Their findings revealed that TD independently influences the prognosis of these individuals. Even for T1-2 patients with TD, postoperative adjuvant chemotherapy should be considered. The research revealed 9 instances of stage I, 81 occurrences of stage II, and 485 cases of stage III based on the original TNM-stage classification. Out of these, 336 cases received adjuvant chemotherapy after surgery, while 239 cases did not. Among the stage I patients, four received adjuvant chemotherapy, while 234 stage II–III patients did not. The nomogram analysis shows that postoperative adjuvant chemotherapy is the only protective factor for patients with TD-positive GC. Therefore, we propose that for GC patients with TD, regardless of their specific staging, as long as the patient’s general condition can tolerate chemotherapy, postoperative adjuvant chemotherapy is recommended to improve the survival rate and prognosis.

There are several limitations in this study: (1) This study was a single-center retrospective analysis, and the constructed model lacked external validation although it performed well for internal validation. In follow-up, we intend to conduct a multi-center, large-sample prospective study to validate this model. (2) When constructing the model, we encountered difficulties in constructing a nomogram based on each grade of pT-stage and pN-stage. This suggests that the degree of metastasis of the tumor (neural invasion, number of TD) has a more important prognostic value than the primary tumor T-stage and N-stage in TD (+) GC patients. (3) The accurate counting of time to tumor recurrence and site of recurrence was challenging, therefore disease-free survival time (DFS) statistical analysis was not performed.

## Conclusions

In this study, a nomogram for determining the prognosis of patients with TD-positive GC was successfully constructed. The nomogram showed a greater positive net gain compared to the eighth edition of the TNM stage, indicating its clinical value. The impact of TD on TD-positive GC’s prognosis should be taken into account not just based on the quantity of TD, but also on gastric cancer’s tumor stage. While there is no direct evidence suggesting that GC patients with stage I combined TD specifically benefit from postoperative adjuvant chemotherapy, overall data has demonstrated that postoperative adjuvant chemotherapy is a protective factor for patients with TD-positive GC. Therefore, it is recommended that patients with TD-positive GC receive postoperative adjuvant chemotherapy, irrespective of their stage, as long as their physical condition allows.

## Supplemental Information

10.7717/peerj.17751/supp-1 Supplemental Information 1 Raw data

10.7717/peerj.17751/supp-2 Supplemental Information 2 Tripod checklist

## Abbreviations

TD: Tumor deposits
GC: Gastric cancer
AJCC: American Joint Committee on Cancer
UICC: Union for International Cancer Control
OS: Overall survival
DFS: Disease-free survival
AUC: Area under curve
DCA: Decision curve analysis

## Additional Information and Declarations

### Data Availability

The following information was supplied regarding data availability: The raw measurements are available in the Supplementary Files.
